# Shifts in the Bacterial Population and Ecosystem Functions in Response to Vegetation in the Yellow River Delta Wetlands

**DOI:** 10.1128/mSystems.00412-20

**Published:** 2020-06-09

**Authors:** Jianing Wang, Jingjing Wang, Zheng Zhang, Zhifeng Li, Zhiguo Zhang, Decun Zhao, Lidong Wang, Feng Lu, Yue-zhong Li

**Affiliations:** a State Key Laboratory of Microbial Technology, Institute of Microbial Technology, Shandong University, Qingdao, People’s Republic of China; b School of Life Science, Shandong University, Qingdao, People’s Republic of China; c National Nature Reserve Administration of Yellow River Delta, Dongying, People’s Republic of China; California Department of Water Resources

**Keywords:** soil bacterial composition, *Cyanobacteria*, *Gemmatimonadetes*, omics analyses, carbon cycling, estuarine coastal wetland, Yellow River Delta

## Abstract

Vegetation represents probably the most crucial step for the ecosystem functions of wetlands, but it is unclear how microbial populations and functions shift along with vegetation. In this study, we found that the richness and diversity of soil bacteria increased with vegetation levels and that the community composition was distinctly shifted from bare to vegetative places. The bare land displayed an extremely high abundance of *Cyanobacteria* as a monospecies genus, while a *Gemmatimonadetes* genus was predominant as multiple species in all the vegetative wetlands, suggesting their important ecosystem functions and potential mechanisms. Expression of the genes related to photosynthesis was enriched exclusively in bare land. Genes involved in biological organic carbon metabolism and the cycling of main elements (C, N, S, and P) were highly expressed in vegetative wetlands and were mostly included in the metagenome-assembled genome (MAG) of *Gemmatimonadetes*. Some compounds identified from soil metabolomic results also corresponded to pathways involving these key active genes. *Cyanobacteria* is thus responsible for the carbon sink in early infertile wetlands, and *Gemmatimonadetes* plays a crucial role in ecosystem functions in vegetative wetlands. Our results highlight that the soil microbial populations execute ecosystem functions for wetlands and that vegetation is the determinant for the population and functional shifts in the coastal estuarine wetland of the Yellow River Delta.

**IMPORTANCE** Vegetation probably represents the most crucial step for the ecosystem functions of wetlands, but it is unclear how microbial populations and functions shift in pace with the colonization and succession of vegetation. In this study, we found that a *Cyanobacteria* monospecies genus and a *Gemmatimonadetes* multispecies genus are fastidiously predominant in the bare and vegetative wetlands of the Yellow River Delta, respectively. Consistently, photosynthesis genes were enriched exclusively in bare land, while genes involved in biological organic carbon metabolism and the cycling of main elements were highly expressed in vegetative wetlands, were mostly included in the MAG of *Gemmatimonadetes*, and were consistent with soil metabolomic results. Our results provide insight into the adaptive succession of predominant bacterial species and their ecosystem functions in response to the presence of vegetation.

## Abstract (precis)

Vegetation probably represents the most crucial step for the ecosystem functions of wetlands, but it is unclear how microbial populations and functions shift in pace with the colonization and succession of vegetation. In this study, we found that a *Cyanobacteria* monospecies genus and a *Gemmatimonadetes* multispecies genus are fastidiously predominant in the bare and vegetative wetlands of the Yellow River Delta, respectively. Consistently, photosynthesis genes were enriched exclusively in bare land, while genes involved in biological organic carbon metabolism and the cycling of main elements were highly expressed in vegetative wetlands, were mostly included in the MAG of *Gemmatimonadetes*, and were consistent with soil metabolomic results. Our results provide insight into the adaptive succession of predominant bacterial species and their ecosystem functions in response to the presence of vegetation.

## INTRODUCTION

Rivers with large sediment loads contribute yearly approximately 7 × 10^9^ tons of sediments to the ocean (1–3). The fluvial sediments are deposited mainly in the estuary and develop into coastal estuarine delta wetlands, playing an important role as an ecosystem for global bioremediation procedures (4–7). Environments are able to shape the embedded microbial communities, which, as the key factors in a wide range of biogeochemical cycles, in turn mold environmental characteristics and functions (8). Many studies have examined the wetland microbiota and their changes, for example, the microbial community structures and their shifts in salinity gradients of the Baltic Sea (9), the Delaware Bay (10), or the Yangtze River estuary (11). Some additional studies also explored the ecosystem functions of wetland microbiota (12–15). Those studies mostly mapped the overall or specific microbiota, predominantly from the 16S rRNA gene sequence analysis, but provided little information for the characterization of their ecosystem functions in wetlands.

The Yellow River, which is the second largest river in China, is regarded as the world’s largest contributor of fluvial sediment load to the ocean (1–3). Approximately 30% to 40% of the sediment transported by the Yellow River is deposited in an estuary, forming the Yellow River Delta, which covers an area of 7,870 km^2^ with average deposition thickness around 15 m (2, 16). Due to the continuous sediment deposit in the estuary, the Yellow River Delta is expanding toward the ocean. The Yellow River Delta is a typical water-limited area, with annual average rainfall of 590.9 mm and potential evaporation of 1,962 mm (17, 18). Furthermore, due to climate change and human activities, water discharge and sediment load of the Yellow River are being dramatically reduced (19, 20). The soil salinity in the region is in a wide range from 0.11 to 10.50 dS m^−1^, and the salinity in topsoil is generally higher than that in subsoil (21).

Vegetation represents probably the most crucial step for delta wetlands to develop ecosystem functions. On the basis of canonical correspondence analysis (CCA) results, Cui et al. revealed that plant species and vegetation community are mainly influenced by soil salinity and water depth (22). Vegetation planting in the Yellow River Delta goes through a natural process of succession consisting of newborn bare land, the pioneering Suaeda salsa, the reeds of Phragmites australis, and then the other vegetation types such as Tamarix chinensis, Imperata cylindrica, Apocynum venetum, etc., which form clear vegetation belts, roughly in the pattern “bare land—Suaeda salsa—Phragmites australis—other vegetation types” along the direction away from the ocean (12, 18, 21). The Yellow River Delta is thus an ideal ecosystem for investigations of the structural and functional shifts in microbial communities in response to vegetation. Microbes in the Yellow River Delta have been widely investigated, for example, in studies of the bacterial distribution patterns between water and sediment (23, 24), the effects of soil salinity and petroleum contamination on soil bacterial community structures (25, 26), the diversity and distribution of *nirK*-harboring denitrifying bacteria in the water column (27), and nitrite-dependent anaerobic methane-oxidizing bacteria in sediments (28). The bacterial community structures in wetland soil, from saline bare land to the marshy grassland of P. australis (12, 29), as well as in different tidal flats (30) have also been investigated. Those previous studies revealed that the bacterial compositions differed significantly along vegetative succession regions, and the dominant phyla across all samples were *Proteobacteria*, *Bacteroidetes*, and *Firmicutes*. The microbial diversity was lower in bare land than at the other sites, and the microbial communities were significantly correlated with salinity gradient, vegetation, and soil organic matter. However, how the microbial communities and functions shift in pace with the colonization and succession of vegetation and what the microbial ecological functions are in the bare and vegetative wetlands are still unclear. The reciprocity of microbiota and plantation as well as their functions requires deep and comprehensive analyses.

This study aimed to investigate the characteristics of microbial communities and their functional changes in bare and vegetative wetlands. We collected soil samples along the early vegetation transition zones in the Yellow River Delta wetland. We found that bacterial compositions were significantly shifted from bare to vegetative places; a monospecific *Cyanobacteria* genus and a multiple-specific *Gemmatimonadetes* genus were fastidiously predominant in the bare and the vegetative wetlands, respectively. To understand the ecosystem functions and potential functional mechanisms of wetland bacteria, we performed metatranscriptomic sequencing and metabolomic identification of bare and vegetative soil samples. In addition, we annotated a metagenome-assembled genome (MAG) of *Gemmatimonadetes* binned from metagenomic sequences. The omics data showed that photosynthesis genes, probably from *Cyanobacteria*, were highly expressed in bare land, while genes involved in biological organic carbon (OC) metabolism and the cycling of main elements, mostly found in the MAG of *Gemmatimonadetes*, were more active in vegetative wetlands. Our results provide insight into the soil development of newborn wetlands and the adaptive shift in bacteria and their ecosystem functions in response to vegetation in the Yellow River Delta wetlands.

## RESULTS

### Biogeographic separation of bacteria in bare and vegetative wetlands.

**FIG 1:**
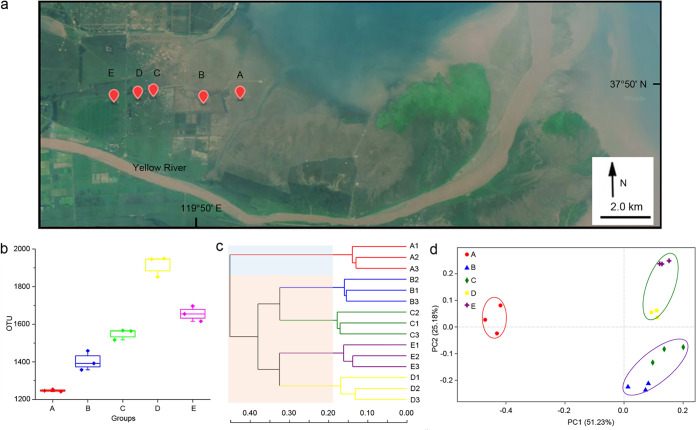
Biogeography characteristics of the bacterial communities in bare and vegetative wetlands of the Yellow River Delta. (a) The geographical sampling locations. Plot A is bare land near the ocean at a distance of ∼2.1 km; plots B and C (at a distance of 2.2 km; the distance between plots A and B is 1.8 km) have sparse Suaeda salsa; plot D (0.7-km distance from plot C) is densely grown with Suaeda salsa; and plot E (1.2-km distance from plot D), which is the sampling site farthest from the ocean, is a grassland of Phragmites communis reeds. Three samples at each plot were collected from different places within a distance of approximately 3 to 5 m (the geographical information and the geomorphology refer to Fig. S1a). Background image was acquired freely from http://www.gscloud.cn/. (b) OTU (97% similarity) numbers in the five plots. (c) Hierarchical clustering tree based on the Bray-Curtis metric at the OTU level. (d) PCoA of OTUs with weighted UniFrac distances. The PERMANOVA data are as follows: for plot A versus plots B/C, *F* = 12.94, *P* = 0.014; for plot A versus plots D/E, *F* = 10.93, *P* = 0.013; for plots B/C versus plots D/E, *F* = 7.00, *P* = 0.003.

To investigate the microbial compositions in the Yellow River Delta wetlands, we collected 15 soil samples at five plots ranging from near the ocean to distant from the ocean (Fig. 1a). In this study, we focused on the early pedogenetic stage of wetland, and the sampling sites were at newborn barren land and wetlands planted with pioneering halophytes: i.e., plot A is barren land; plots B and C are wetlands with sparse Suaeda salsa; plot D has densely grown S. salsa; and plot E is at Phragmites australis grassland. According to the ecohydrological data (18), plot A was in the supratidal zone (normally not affected by tides, except the high tide), while other plots were all at locations away from tides. We performed high-throughput sequencing on the 16S rRNA gene amplicons to evaluate the bacterial compositions in these 15 soil samples. After filtering, we obtained 696,445 valid 16S rRNA gene fragments with an average read length of 440.58 bp. In total, 3,986 bacterial operational taxonomic units (OTUs) (at the 97% similarity level) were revealed from these sequences. The Good’s coverage, an estimator of sampling completeness, ranged from 95.84% to 97.61% for the 15 samples, which indicated that the data set was adequate for the estimation of bacterial diversity.

Comparisons of the data showed that the bacterial OTU numbers were highly similar in the three sympatric samples but differed in the different plots (Fig. 1b). The bare land soil (plot A) had the lowest OTU numbers (1,247.667 ± 6.028), which increased to 1,915.667 ± 55.157 in plot D and then decreased in the P. australis grassland soil (plot E). We calculated the alpha diversity indices for each of the soil samples at the 97% OTU level. Similarly, the bacterial richness and diversity both increased from plot A to plot E but had the highest values at plot D (Fig. S1b). We clustered the bacterial OTUs based on the Bray-Curtis algorithm (Fig. 1c) and principal-coordinate analysis (PCoA) performed with weighted UniFrac distances (total support of PC1 and PC2, 76.41%; Fig. 1d). Interestingly, the bacterial compositions of the five plots were obviously classified into two distinct groups: the bare land group (plot A) and the vegetative wetland group (plots B, C, D, and E). Within the vegetative wetland group, the bacterial compositions was further divided into two subgroups: sparse plantation plots B/C and dense plantation plots D/E. Accordingly, the soil bacterial richness and diversity both increased with the development of vegetation, and the bacterial distribution was obviously associated with vegetation. Interestingly, plot A had the highest number of unique OTUs (363; the relative abundance was 19.28%), while the numbers of OTUs that were exclusively present in the other four vegetative plots were 157 (5.33% in plot B), 176 (3.35% in plot C), 121 (4.04% in plot D), and 223 (6.13% in plot E), respectively (Fig. S2). Only 349 of the OTUs were shared by all five plots, accounting for 35.59%, 32.10%, 36.60%, 35.09%, and 27.40% in plots A to E, respectively (8.76% of the total 3,986 OTUs). Thus, many bacteria in the bare wetland disappeared, and many others appeared in pace with vegetation colonization and succession, which might reflect the functional discrepancies of bacterial communities.

10.1128/mSystems.00412-20.1 FIG S1 Characteristics of plant and soil bacterial alpha diversity in bare and vegetative wetlands of the Yellow River Delta. (a) Close-up views of the sampling sites in the Yellow River Delta. (b) Alpha diversity indices (Ace, Chao1, Shannon, and Simpson) of the plot of bacterial OTUs (97% similarity) from three soil samples at five locations along the vegetation transition zone in the Yellow River Delta. Download FIG S1, TIF file, 2.2 MB. Copyright © 2020 Wang et al. 2020 Wang et al. This content is distributed under the terms of the Creative Commons Attribution 4.0 International license.

10.1128/mSystems.00412-20.2 FIG S2 Venn diagram of the OTUs (97% similarity) from the five wetland plots of the Yellow River Delta. Download FIG S2, TIF file, 0.4 MB. Copyright © 2020 Wang et al. 2020 Wang et al. This content is distributed under the terms of the Creative Commons Attribution 4.0 International license.

We assayed the physicochemical features of the plot soil samples (details are listed in Table S1). The soil pH gradually decreased from 8.79 ± 0.15 at plot A to 7.75 ± 0.08 at plot E, while the soil organic carbon (SOC) level increased from 16.28 ± 4.32 g/kg (plot A) to 39.15 ± 8.47 g/kg (plot E). The levels of salinity content were comparably low in the ocean-close wetlands (plots A and B) but high in the soil far from the ocean (plots C, D, and E), which is mostly consistent with the contour map representing the spatial distribution of soil salinity in coastal zone of the Yellow River Delta surveyed by Yu et al. (21). As results of redundancy analysis (RDA) and a Monte Carlo permutation test showed (Fig. S3), pH and soil water content and salinity were the major factors influencing community composition. The results were rather similar to those reported from previous studies conducted in the Yellow River Delta (12, 29, 30); i.e., the bacterial community near the ocean had the lowest alpha-diversity among the sites, and salinity had an important effect on the microbial community structure.

10.1128/mSystems.00412-20.3 FIG S3 (a and b) Redundancy analysis (RDA) of bacterial communities and environmental parameters at the phylum (a) and family (b) levels. (c and d) Values representing results of the Monte Carlo permutation test performed at the phylum or family level for each physicochemical factor are provided in panels c and d. Download FIG S3, TIF file, 0.6 MB. Copyright © 2020 Wang et al. 2020 Wang et al. This content is distributed under the terms of the Creative Commons Attribution 4.0 International license.

10.1128/mSystems.00412-20.5 TABLE S1 Geochemical features of 15 soil samples collected from five locations along the vegetation transition zone in the Yellow River Delta. Download Table S1, XLSX file, 0.01 MB. Copyright © 2020 Wang et al. 2020 Wang et al. This content is distributed under the terms of the Creative Commons Attribution 4.0 International license.

### Shift in bacterial taxonomies from bare to vegetative wetlands.

**FIG 2:**
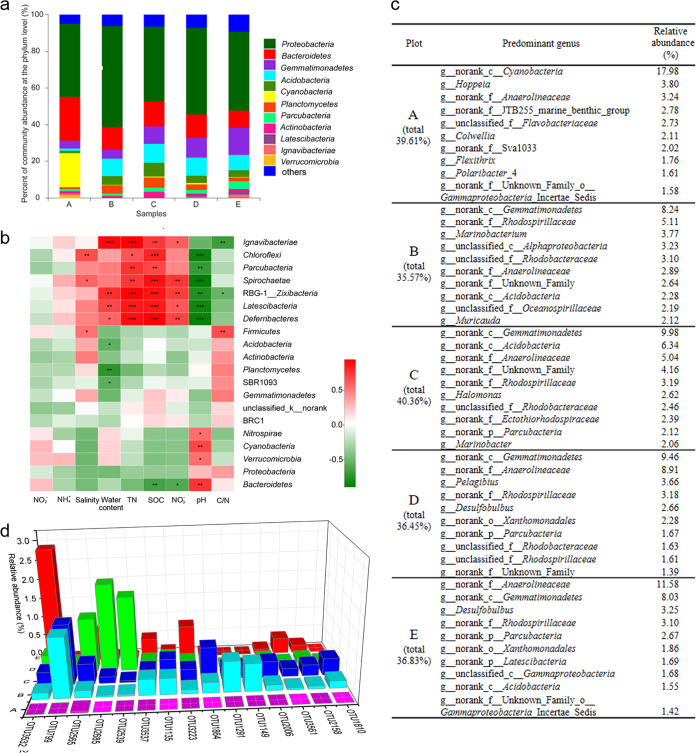
Bacterial community compositions and their responses to environmental factors in wetlands along the vegetation transition zones in the Yellow River Delta. (a) Bacterial community compositions at the phylum level (“others” = <0.05) of the five plots. (b) Pearson correlation heat map of environmental factors and the relative abundances of the 20 most abundant bacterial phyla in wetland soil samples (*, *P* < 0.05; **, *P* < 0.01; ***, *P* < 0.001). (c) Top 10 predominant bacterial genera in five plots. (d) Relative abundances of the 15 most abundant OTUs of g__norank_c__*Gemmatimonadetes* in the five plots.

To investigate the shift of bacterial taxonomies from bare to vegetative wetlands, we annotated the bacterial species for the OTUs based on data from the Silva 16S rRNA gene database at the 70% confidence level, which allows more sequences to be annotated with relatively high accuracy at low taxonomic levels (31, 32). There were 46 phyla, 188 classes, 237 orders, 404 families, and 697 genera in the total of 3,896 OTUs. Consistent with previous results (12, 29), the most predominant phylum was *Proteobacteria*, followed by *Bacteroidetes*, in each of the five wetland plots (Fig. 2a). Notably, the *Cyanobacteria* phylum was present at an extremely high abundance in the soil samples at plot A (18.69%) but was present at a low ratio in all the vegetative plot samples, i.e., 0.28% in plot B, 0.49% in plot C, 0.73% in plot D, and 0.12% in plot E. In contrast, the *Gemmatimonadetes* phylum was relatively abundant in all the vegetative wetlands (9.58% in plot B, 10.38% in plot C, 9.69% in plot D, and 8.21% in plot E) but was low in abundance in the bare wetland (1.30%). Pearson correlation tests showed that the levels of some phyla were strongly correlated with the assayed geochemical variables, while some others had almost no correlation (Fig. 2b).

The predominant genera in plot A were mostly different from those that were predominant in the four vegetative wetlands, in which many abundant bacteria were shared (Fig. 2c shows the top 10 predominant bacterial genera in the five plots). In plot A, an unidentified genus (g__norank_c__*Cyanobacteria*) had the highest relative abundance (17.98%), occupying 95.43% of the total reads of this phylum. Similarly, g__norank_c__*Gemmatimonadetes* was almost always the most abundant genus in the four vegetative regions, occupying 92.91% of the total *Gemmatimonadetes* sequences (Table S2 shows the predominant bacterial community composition at the different taxonomic levels in each plot). The correlations of the two genera *Cyanobacteria* and *Gemmatimonadetes* with the environmental factors were similar to those at the phylum level; i.e., abundance of the *Cyanobacteria* genus was positively correlated only with the pH value (*P* < 0.01), while that of the *Gemmatimonadetes* genus had no positive or negative correlations with the assayed environmental factors (Fig. S4).

10.1128/mSystems.00412-20.4 FIG S4 Pearson correlation heat map of environmental factors and relative abundances of the 20 most abundant bacterial genera in wetland soil samples (*, *P* < 0.05; **, *P* < 0.01; ***, *P* < 0.001). Download FIG S4, TIF file, 0.5 MB. Copyright © 2020 Wang et al. 2020 Wang et al. This content is distributed under the terms of the Creative Commons Attribution 4.0 International license.

10.1128/mSystems.00412-20.6 TABLE S2 Predominant bacterial community compositions in each plot at the different taxonomic levels. Download Table S2, XLSX file, 0.02 MB. Copyright © 2020 Wang et al. 2020 Wang et al. This content is distributed under the terms of the Creative Commons Attribution 4.0 International license.

In total, 31 OTUs were annotated as belonging to the *Cyanobacteria* phylum, 18 of which were located in g__norank_c__*Cyanobacteria*. Interestingly, the most abundant OTU of the *Cyanobacteria* genus occupied 13.27% of the total reads in plot A, followed by the second most abundant OTU (1.72%) and the third most abundant OTU (1.06%), which suggested that a monospecific OTU of *Cyanobacteria* played major ecological functions in the bare land. The *Gemmatimonadetes* phylum contained 137 OTUs, 112 of which belonged to the predominant *Gemmatimonadetes* genus, but the relative abundances of these single-genus OTUs differed significantly in different plots (Fig. 2d). This result suggested that multiple species of the *Gemmatimonadetes* genus were present in the vegetative wetlands.

### Ecosystem functions of bacteria in bare and vegetative wetlands.

**FIG 3:**
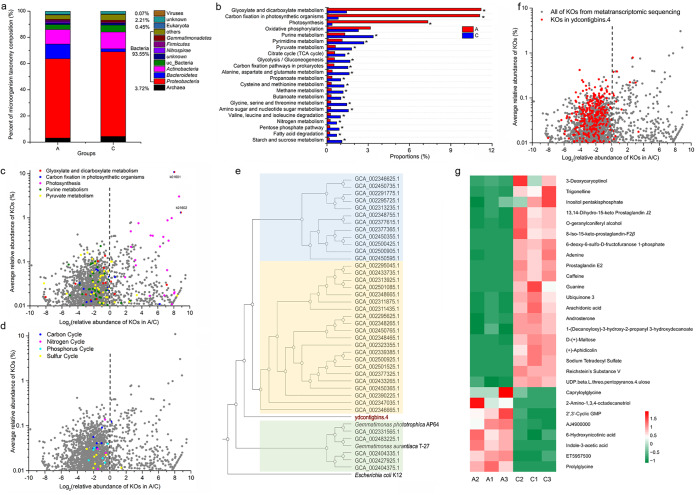
Omics analyses of bacterial functions in bare and vegetative wetlands. (a) Relative abundances of microorganisms (bacteria, archaea, eukaryotes, and viruses) in plots A and C based on metagenomic sequencing. (b) The relative abundances of metabolic pathways of plots A and C at KEGG level 3 based on metatranscriptomic sequencing. Each asterisk (*) indicates a fold change value of >2. (c) Volcano plots of the metatranscriptomics-identified genes associated with the main metabolic pathways in plots A and C. (d) Volcano plots of the metatranscriptomics-identified genes associated with the cycling of elements in plots A and C. (e) A phylogenomic tree of MAGs and cultured genomes of the phylum *Gemmatimonadetes*. (f) Expression patterns of the genes from ydcontigbins.4 in the metatranscriptome. (g) Heat map of the 28 differentially enriched metabolites in the samples of plots A and C using Z-scores [*Z* = (*X* − average)/standard deviation].

To investigate the relationships between the microbial compositions and their ecological functions, we performed metagenomic and metatranscriptomic analyses on the soil samples of plot A (bare wetland) and plot C (as the representative of vegetative wetlands). On the basis of the FastQC report, the Q20 parameter values were >90%, which indicated that the sequencing data sets were of high quality. The clean reads occupied >93% of the raw data (Table S3). The metagenomic sequence data set showed a profile of the present organisms in the soil samples, i.e., bacteria were absolutely predominant in the wetland samples, occupying 93.5% of the total metagenomic sequences, followed by 3.72% archaea, 0.45% eukaryotes, and 0.07% viruses, with 2.21% unknown resources (Fig. 3a), but the data set was insufficient for detailed description of taxonomic abundance at low taxonomic levels. Nevertheless, the metagenomic sequences suggested that bacteria played the major role in microbial ecosystem functions in both the bare and vegetative wetlands.

10.1128/mSystems.00412-20.7 TABLE S3 Metagenomic and metatranscriptomic sequencing information from plot A and C. Download Table S3, XLSX file, 0.04 MB. Copyright © 2020 Wang et al. 2020 Wang et al. This content is distributed under the terms of the Creative Commons Attribution 4.0 International license.

We annotated the metatranscriptomic UniGene data to the corresponding functional classifications based on the Kyoto Encyclopedia of Genes and Genomes (KEGG) database. A total of 9,969 KEGG orthologs (KOs) were retrieved and were consolidated into 45 metabolic subsystems at KEGG level 2 and into 437 metabolic pathways at KEGG level 3. Of the 9,969 genes, 1,726 had a relative abundance of >0.01%, 1,194 of which had a high relative abundance in plot C, while 258 were highly abundant in plot A. These differentially expressed genes were mostly involved in 22 metabolic pathways; 18 pathways were upregulated in plot C, while 4 were upregulated in plot A, i.e., “glyoxylate and dicarboxylate metabolism,” “carbon fixation in photosynthetic organisms,” “photosynthesis,” and “oxidative phosphorylation” (Fig. 3b; additional detailed information is provided in Table S4). As the volcano plot shows, the genes involved in photosynthesis were mostly rich in abundance in plot A (pink spots in Fig. 3c; all the spots represent the 1,726 KOs with >0.01% relative abundance). In contrast, the genes involved in “carbon fixation in photosynthetic organisms” (a pathway different from photosynthesis) and “glyoxylate and dicarboxylate metabolism” (red and blue spots, respectively) were highly expressed mostly in plot C, except K01601 and K01602, which were extremely highly expressed in plot A, resulting in higher relative abundances of the two metabolism pathways in plot A. K01601 and K01602, both belonging to the pathways of “carbon fixation in photosynthetic organisms” and “glyoxylate and dicarboxylate metabolism,” are defined as ribulose-bisphosphate carboxylases (large and small chain). Thus, the only highly expressed form of metabolism in plot A was photosynthesis. These photosynthesis-related genes had homologues mostly in single genomes of *Cyanobacteria* species, such as Synechococcus elongatus PCC 7942 (33, 34), which suggested that the predominant *Cyanobacteria* genus was predominantly responsible for photosynthesis in the bare wetland.

10.1128/mSystems.00412-20.8 TABLE S4 Abundance of the genes associated with metabolic pathways and cycling of elements retrieved from the 9,969 KEGG orthologs (KOs). Download Table S4, XLSX file, 0.04 MB. Copyright © 2020 Wang et al. 2020 Wang et al. This content is distributed under the terms of the Creative Commons Attribution 4.0 International license.

Consistent with the KEGG metabolism analysis, the genes for purine metabolism and pyruvate metabolism were expressed mostly at higher levels in plot C than in plot A (green and yellow spots in Fig. 3c). We also observed the genes associated with carbon cycling (not within photosynthesis; 9 KOs), nitrogen cycling (15 KOs), phosphorus cycling (7 KOs), and sulfur cycling (18 KOs) (35–37) among the 9,969 annotated genes (Table S4). Volcano plot analysis showed that almost all of these cycle-associated genes were upregulated in the vegetative wetland (Fig. 3d). Thus, the soil microbial ecological functions of the bare wetlands are different from those of the vegetation-colonized wetlands; highly expressed genes in the bare wetlands were associated mainly with photosynthesis, while the genes involved in the biological metabolism and cycling of main elements (C, N, P, and S) were highly expressed mostly in the vegetative wetlands. We recovered MAGs from about 30 Gb of soil metagenomic DNA. In total, 17 MAGs were obtained, 5 of which were of high quality, with a completeness range of 41.95% to 78.41% and a contamination range of 0% to 6.04%. The MAG of ydcontigbins.4 (completeness, 72.04%; contamination, 6.04%) was taxonomically assigned to *Gemmatimonadetes*. We constructed a phylogenomic tree of ydcontigbins.4 with the known two sequenced genomes and MAGs of *Gemmatimonadetes* (38), which showed that ydcontigbins.4 represented a new kind of *Gemmatimonadetes* (Fig. 3e). Annotation of ydcontigbins.4 using the KEGG database retrieved 377 KOs (Table S5). Interestingly, 362 of the 377 KOs were also identified in the metatranscriptomic KOs, and almost all of the 362 KOs were upregulated in plot C (Fig. 3f). The results strongly suggested that the predominant *Gemmatimonadetes* were highly active in vegetative wetlands of the Yellow River Delta.

10.1128/mSystems.00412-20.9 TABLE S5 Results of annotation of genes from ydcontigbins.4 based on KEGG database. Download Table S5, XLSX file, 0.02 MB. Copyright © 2020 Wang et al. 2020 Wang et al. This content is distributed under the terms of the Creative Commons Attribution 4.0 International license.

**FIG 4:**
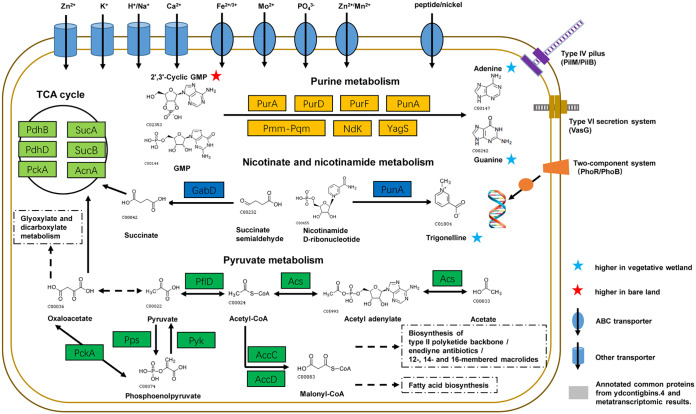
Visualization of proteins identified in both ydcontigbins.4 and metatranscriptomic results and metabolites identified from metabolomics results. TCA cycle components are as follows: SucA, 2-oxoglutarate dehydrogenase E1 component; SucB, 2-oxoglutarate dehydrogenase E2 component; AcnA, aconitate hydratase; PckA, phosphoenolpyruvate carboxykinase; PdhD, dihydrolipoamide dehydrogenase; PdhB, pyruvate dehydrogenase E1 component beta subunit. Purine metabolism components are as follows: PurA, adenylosuccinate synthase; PurD; phosphoribosylamine-glycine ligase; PurF, amidophosphoribosyltransferase; PunA, purine-nucleoside phosphorylase; Pmm-Pgm, phosphomannomutase-phosphoglucomutase; Ndk, nucleoside-diphosphate kinase; YagS, xanthine dehydrogenase YagS FAD-binding subunit. Nicotinate and nicotinamide metabolism components are as follows: PunA, purine-nucleoside phosphorylase; GabD, succinate-semialdehyde dehydrogenase/glutarate-semialdehyde dehydrogenase. Pyruvate metabolism components are as follows: Acs, acetyl coenzyme A (acetyl-CoA) synthetase; PflD, formate C-acetyltransferase P; PckA, phosphoenolpyruvate carboxykinase (ATP); Pps, pyruvate, water dikinase; Pyk, pyruvate kinase; AccC, acetyl-CoA carboxylase, biotin carboxylase subunit; AccD, acetyl-CoA carboxylase carboxyl transferase subunit beta. Type IV pilus components are as follows: PilM, type IV pilus assembly protein PilM; PilB, type IV pilus assembly protein PilB. The type VI secretion system component is as follows: VasG, type VI secretion system protein VasG. Two-component system components are as follows: PhoR, two-component system, OmpR family, phosphate regulon sensor histidine kinase; PhoB, two-component system, OmpR family, alkaline phosphatase synthesis response regulator. Other details are listed in Table S5.

On the basis of the MAG annotation and metatranscriptomic results, we constructed a simple visual diagram of the *Gemmatimonadetes* MAG (Fig. 4). The MAG contained a variety of transporters responsible for Zn^2+^, K^+^, H^+^/Na^+^, and Ca^2+^; ABC transporters for Fe^2+^/^3+^, Mo^2+^, PO_4_^3−^, Zn^2+^/Mn^2+^, and peptide/nickel; the type VI secretion system protein VasG; the type IV pilus assembly protein PilM/PilB; and the two-component system PhoP/PhoB1 associated with phosphate absorption. The diagram also included purine metabolism, pyruvate metabolism, nicotinate and nicotinamide metabolism, and the tricarboxylic acid (TCA) cycle. To investigate the involved potential microbial metabolism, we further performed metabolomic analysis using high-performance liquid chromatography–tandem mass spectrometry (HPLC-MS/MS) on the three soil replicates from plots A and C. We identified 86 compounds (Table S6), 20 of which were present in higher concentrations in the vegetative wetland samples and 8 of which had higher concentrations in the bare land (Fig. 3g). Some of the compounds that were abundant in vegetative wetlands were associated with the upregulated genes of ydcontigbins.4. Interestingly, while the KOs for purine metabolism, pyruvate metabolism, nicotinate and nicotinamide metabolism, and the TCA cycle were identified in the ydcontigbins.4 MAG and at higher expression levels in the vegetative wetland as determined by metatranscriptomics, some associated metabolic products were also identified in the soil metabolomics, although the metabolomics might have involved the metabolites secreted by vegetation in plot C where sparse S. salsa had grown as well as noise from the background soil organic matter. For example, 2′,3′-cyclic GMP (abundant in plot A) is a purine derivative and is converted by purine metabolism (the enzymes were highly expressed in plot C) into adenine (abundant in plot C). Trigonelline was present at a high concentration in vegetative wetlands. The compound is convertible from nicotinamide d-ribonucleotide through the activity of some catalytic enzymes, such as PunA. Succinate and oxaloacetate are important components of the TCA cycle; the former is formed from succinate semialdehyde through the activity of GabD, while the latter is formed from acetate by that of a series of pyruvate metabolism enzymes (Acs, PflD) and was highly expressed in plot C. The omics analyses suggested that *Gemmatimonadetes* played crucial roles in the biological metabolism and cycling of main elements in vegetative wetlands of the Yellow River Delta.

10.1128/mSystems.00412-20.10 TABLE S6 Metabolite information from plots A and C determined using HPLC-MS/MS. Download Table S6, XLSX file, 0.02 MB. Copyright © 2020 Wang et al. 2020 Wang et al. This content is distributed under the terms of the Creative Commons Attribution 4.0 International license.

## DISCUSSION

Coastal estuarine deltas are found at the edge of marine ecosystems, terrestrial ecosystems, and freshwater ecosystems and are thus influenced by many environmental parameters. Vegetation represents probably the most crucial process for various wetlands to develop their ecosystem functions. Oliveira et al. (39) once investigated the effects of salt marsh vegetation on sediment bacterial communities. They found that microbial colonization and organic matter decomposition are enhanced under the influence of salt marsh plants and confirmed that plant coverage is a major determinant for the processes of organic matter recycling in intertidal estuarine sediments. Generally, photosynthetic *Cyanobacteria* occurs widely in fresh, brackish, and salt waters (40), while *Gemmatimonadetes* frequently appears in high relative abundance in different habitats (41, 42). In our results presented in this study, the bare land exhibited extremely high concentrations of *Cyanobacteria* as a monospecies genus, while a single *Gemmatimonadetes* genus was predominant with multiple species in all the vegetative wetlands of the Yellow River Delta. Omics analyses showed that photosynthesis genes, probably from *Cyanobacteria*, were highly expressed in bare land, while the processes of biological organic carbon metabolism and the cycling of main elements, which were mostly present in a *Gemmatimonadetes* MAG, were more active in vegetative wetlands. The results provide some new insights into the characteristics of bacterial community succession in coastal estuary wetlands in pace with vegetation and imply specific and distinct ecosystem functions of *Cyanobacteria* and *Gemmatimonadetes* in wetlands and their potential functional mechanisms.

*Cyanobacteria* acts as a primary producer of carbon and nitrogen in nutrient-poor ecosystems. The bacterium has the capacities for carbon (CO_2_) fixation, nitrogen (N_2_) fixation, sequestration of iron, and storage of many other essential nutrients, thus circumventing nutrient limitations. In mangrove sediments, *Cyanobacteria* was found to be present as a diverse assemblage, with clear differences among mangrove habitats ranging from shore to forest (43). *Cyanobacteria* was also found to appear dominantly in subsurface flow-constructed wetland microcosms without vegetation compared with those planted with P. australis (44). In this study, we found that *Cyanobacteria* was predominant as the monospecies genus g__norank_c__*Cyanobacteria*, which suggested that single monospecific OTUs of *Cyanobacteria* played crucial ecological functions in bare land. Consistently, the upregulated genes in the bare land were related mainly to photosynthesis, probably for the accumulation of organic carbons, which are limited in bare land. The results implied that carbon procurability, in addition to the other soil physicochemical characteristics such as pH and salinity, is also a major driving force for the shift in functional bacteria in bare and vegetative wetlands. The microorganisms that participate in photosynthetic carbon sequestration played a key role in sustainment and function of the microbial communities in bare wetlands. Because the photosynthesis genes were highly consistent with the genome annotation of single *Cyanobacteria* species (33, 34) and a monospecific *Cyanobacteria* predominantly occurred in bare land, we imagined that the accumulation of organic carbons was accomplished mainly by single-species cells.

In contrast, genes involved in biological organic carbon metabolism and the cycling of main elements (C, N, P, and S) were enriched in the vegetative wetland, which suggested that the microorganisms that participate in these processes played roles in the vegetation-colonized wetlands. *Gemmatimonadetes* was predominant in all the vegetative wetlands of the Yellow River Delta. Although *Gemmatimonadetes* has been frequently detected in various habitats, only a few species have been identified. The type species of *Gemmatimonadetes*, Gemmatimonas aurantiaca T-27, was isolated from a laboratory-scale enhanced biological phosphorus removal (EBPR) reactor (45). The bacterium is able to convert soluble phosphorus into insoluble phosphorus and thus accumulate a significant amount of polyphosphate inside the cells (45, 46). G. aurantiaca and other *Gemmatimonadetes* species are considered important organisms that are relevant to phosphorus removal from wastewater (47). Our genomic analysis of ydcontigbins.4, an MAG from the Yellow River Delta wetland of *Gemmatimonadetes*, showed that this bacterium harbors a number of genes associated with the transport of a variety of ions, including phosphate, as well as a two-component system related to phosphate absorption (PhoR/PhoB) (48). The predominance of *Gemmatimonadetes* in vegetative wetlands suggested that the bacterium had potential ecological functions in changing the physical-chemical properties and enhancing the cycling of major elements in wetlands. In addition, the *Gemmatimonadetes* MAG harbors a variety of genes associated with biological organic carbon metabolism and transport of a variety of ions, suggesting its important roles in vegetative wetlands, as confirmed by metatranscriptomic and metabolomic analyses. Because multiple specific *Gemmatimonadetes* fastidiously occurred in vegetative wetlands and changed in different vegetative places, we suggested that the processes of biological organic carbon metabolism and the cycling of main elements in soil were accomplished by cooperation of different microbes, including different OTUs of *Gemmatimonadetes*. The shift in microbial communities and the expression patterns of the ecofunctional genes were determined by vegetation planting or its absence.

Coastal estuarine deltas provide humans with critical ecological services and economic benefits (49, 50). In recent decades, almost all river deltas around the world have been impacted by human activities and by the increased frequency and severity of extreme runoff events that have occurred as a consequence of climate variability and change (17). Microorganisms play crucial roles in the bioremediation of deltaic wetland ecosystems, and elucidation of microbial biogeography and their structural and functional shifts is important. Different ecosystems gestate localized microbial communities, which not only play different ecological functions for the habitats but also are susceptible to surrounding environmental factors. The microbial community structure was distinctly shifted from bare to vegetative wetlands but changed in vegetative wetlands in accordance with the succession of the types and density. As such, coverage of different plants on wetlands might similarly provide efficient ecosystem functions.

## MATERIALS AND METHODS

### Sample collection.

The soil samples were collected from five plots (i.e., plots A to E) in the Yellow River Delta along the vegetation transition zone on 25 December 2017 (Fig. 1a). We collected three soil samples of the 5-to-10-cm layer from different places at each plot. All of the samples were placed in sterilized bags and transferred to the laboratory at low temperatures within 6 h. The soil samples were separated into two parts: one was air-dried and sieved through a 2-mm-pore-size sieve for physicochemical analysis, and the other was stored at –80°C for further omics analyses.

### Soil geochemical analysis.

Soil samples were air-dried and sieved through a 2-mm-pore-size sieve. The soil salinity and pH were determined by mixing the dried soil with deionized water (free of CO_2_) at a ratio of 1:5 (wt/vol) and measured by using a DDB-350F microprocessor conductivity meter (INESA Scientific Instrument Co., Ltd., Shanghai, China). The (percent) water content in soil was assayed by oven-drying samples at 105°C. The SOC and total N (TN) contents were measured with a T/N analyzer (Multi N/C 3100; Analytik Jena, Jena, Germany). Nitrate (NO_3_^−^), nitrite (NO_2_^−^), and ammonium (NH_4_^+^) concentrations in soil were determined as previously described (51).

### Sequencing and analysis of the 16S rRNA gene.

DNA was extracted from soil samples by using a FastDNA spin kit for soil (MP Biomedicals, LLC, USA) according to the manufacturer’s instructions. A NanoDrop instrument and 1% agarose electrophoresis were used to determine the DNA concentration and quality. The V3–V4 hypervariable region sequences were amplified by using the primers 338F (5′-ACTCCTACGGGAGGCAGCAG-3′) and 806R (5′-GGACTACHVGGGTWTCTAAT-3′). PCRs were conducted with a thermocycler PCR system (GeneAmp 9700; ABI, USA) as follows: predenaturation at 95°C for 3 min; 27 cycles of 95°C for 30 s, 55°C for 30 s, and 72°C for 45 s; and a final extension at 72°C for 10 min. The PCR products were gel purified using an Omega gel extraction kit (Omega, USA), normalized to equimolar amounts, and sequenced using 2 × 300-bp chemistry on a MiSeq platform at Majorbio Bio-pharm Technology Co., Ltd. (Shanghai, China).

The sequence data were processed using QIIME2 (52). Raw fastq files were quality filtered through the use of Trimmomatic (53) and merged by the use of FLASH (54). Chimeric sequences were identified and removed using UCHIME (55). OTUs were clustered with a 97% similarity cutoff using UPARSE (56). Due to the differences in sequencing depth, we normalized the number of OTUs to the minimum observed across all samples. The taxonomy of each representative OTU sequence was analyzed by the use of the Ribosomal Database Project (RDP) Classifier algorithm against the Silva 16S rRNA gene database (release 128) with a 70% confidence threshold.

### Metagenomic and metatranscriptomic sequencing and analysis.

Metagenomic libraries were prepared using a soil DNA and Illumina Nextera XT kit (Illumina, USA) according to the manufacturer’s protocol. To prepare the metatranscriptomic libraries, soil total RNA was extracted using an RNA PowerSoil total RNA isolation kit (MoBio, USA). After removal of residual DNA and rRNA, the mRNA-enriched RNA was converted to cDNA. The cDNA libraries were constructed using an NEBNext Ultra Directional RNA library prep kit for Illumina sequencing (Illumina, USA).

Raw sequencing reads were checked for potential sequencing issues and contaminants using FastQC. Clean reads were obtained by removing low-quality, N-rich, and adapter-polluted reads from the raw reads using Trimmomatic (53). Assembly softwares Megahit (metagenome) (57) and Trinity (metatranscriptome) (58) were used to assemble clean reads to UniGene data. Metatranscriptomic UniGene data were annotated against the KEGG database for gene functions. Values representing numbers of transcripts per million (TPM) were calculated by the use of RSEM (59) to analyze gene expression. The KEGG pathway enrichment analyses were conducted based on TPM. MAGs were recovered from the metagenomic sequences using MetaBAT (60) and further annotated using the KEGG Automatic Annotation Server (KAAS) (61). The phylogenomic tree of MAGs and cultured genomes was analyzed using CVtree (62).

### Soil metabolomic analysis.

Soil metabolomic analysis was performed using HPLC-MS/MS. Fresh soil samples were immediately frozen in liquid nitrogen, freeze-dried, and then ground to powder. Approximately 100 mg of each sample was combined with 0.5 ml of 80% aqueous methanol (0.1% formic acid), and the mixture was shaken well, placed on ice for 5 min, and then centrifuged at 15,000 rpm for 10 min at 4°C. The supernatant was diluted to 60% aqueous methanol, and then the samples were filtered using a 0.22-μm-pore-size membrane.

The soil metabolic profiles were determined on a Vanquish ultra-high-performance liquid chromatography (UHPLC) system (Thermo Scientific, USA) coupled with a QE-HF-X mass spectrometer (Thermo Scientific, USA). A Hyperil Gold column (C_18_) was used at 40°C for chromatographic separation. The mobile phase included the use of solvent A1 (positive-ion mode; LC-grade pure water with 0.1% formic acid) or solvent A2 (negative-ion mode; LC-grade pure water with 5.0 mM ammonium acetate, pH 9.0) and solvent B (methanol) at a rate of 0.2 ml/min. The solvent B gradient was changed as follows: 2.0% (0 to 1.5 min), 2.0% to 100% (1.5 to 12 min), 100% (12 to 14 min), and 2.0% (14 to 16 min). The mass spectrometer was operated in positive-/negative-polarity mode with a spray voltage of 3.2 kV, capillary temperature of 320°C, sheath gas flow rate of 35 arb (arbitrary units, a specific unit of Thermo Fisher MS instruments), and aux gas flow rate of 10 arb.

The raw data files generated by UHPLC-MS/MS were processed using Compound Discoverer (Thermo Fisher) to perform peak alignment, peak picking, and quantitation for each metabolite. The main parameters were set as follows: retention time tolerance, 0.2 min; actual mass tolerance, 5 ppm; signal intensity tolerance, 30%; signal/noise ratio, 3; and minimum intensity, 100,000. Peak intensities were normalized to the total spectral intensity. The normalized data were used to predict the molecular formula based on additive ions, molecular ion peaks, and fragment ions. Peaks were matched with the mzCloud and ChemSpider databases to obtain accurate qualitative and relative quantitative results.

### Statistical analysis.

Alpha diversity indices (Ace, Chao1, Shannon, and Simpson), Bray-Curtis distances, and weighted UniFrac distances were calculated using scripts in QIIME (52). Hierarchical clustering tree construction and principal-coordinate analysis (PCoA) were conducted using Bray-Curtis distances and weighted UniFrac distances, respectively. The differences between the microbial communities of bare wetland (plot A), sparse plantation (plots B/C), and dense plantation (plots D/E) were tested using permutational multivariate analysis of variance (PERMANOVA) (R: vegan package). Redundancy analysis (RDA) was performed to identify the relationship between environmental parameters and bacterial community composition with Canoco. A Monte Carlo permutation test (499 permutations with unrestricted permutation) was performed to investigate the statistical significance of relationships between taxon (phylum and family) data and environmental parameters. A Pearson correlation heat map was constructed to determine the relationships between bacterial communities and environmental variables on the phylum and genus levels (R: pheatmap package).

### Data availability.

All of the sequencing raw data have been deposited in the NCBI Sequence Read Archive (SRA) database under the accession numbers PRJNA541239, PRJNA550167, and PRJNA553262. The metabolomics data have been deposited in the figshare database (https://doi.org/10.6084/m9.figshare.12117555.v1).
