# Supplementary figures and images for: Shifts in the Bacterial Population and Ecosystem Functions in Response to Vegetation in the Yellow River Delta Wetlands
Source: mSystems. 2020 Jun 9;5(3):e00412-20. doi: 10.1128/mSystems.00412-20 (PMC7289592; doi:10.1128/mSystems.00412-20)

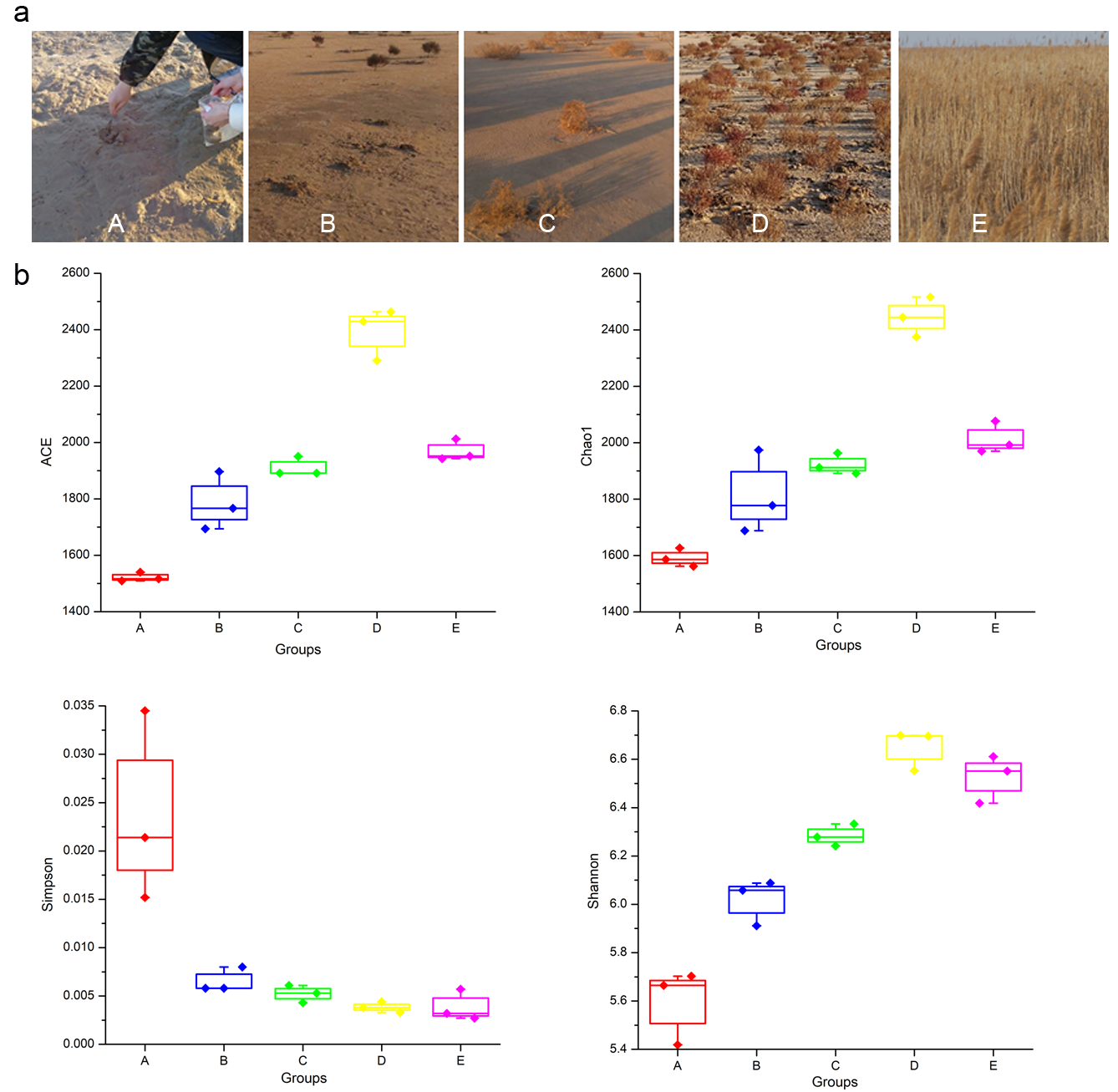

Supplement: FIG S1 [file mSystems.00412-20-sf001.tif]

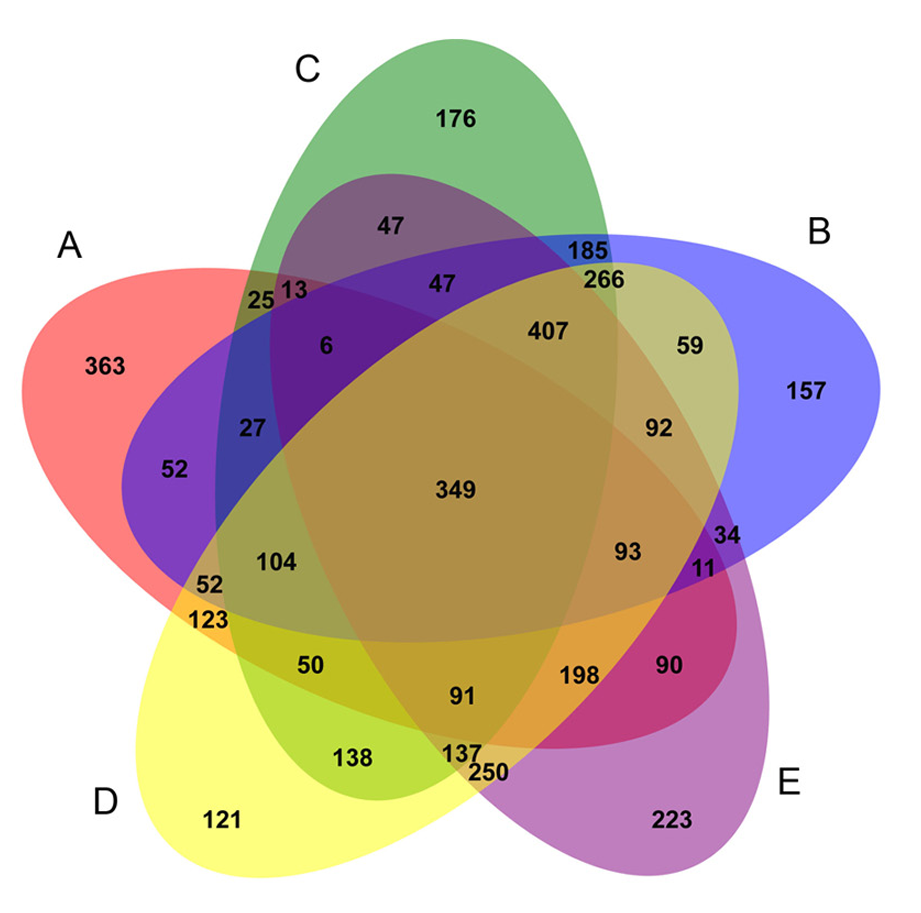

Supplement: FIG S2 [file mSystems.00412-20-sf002.tif]

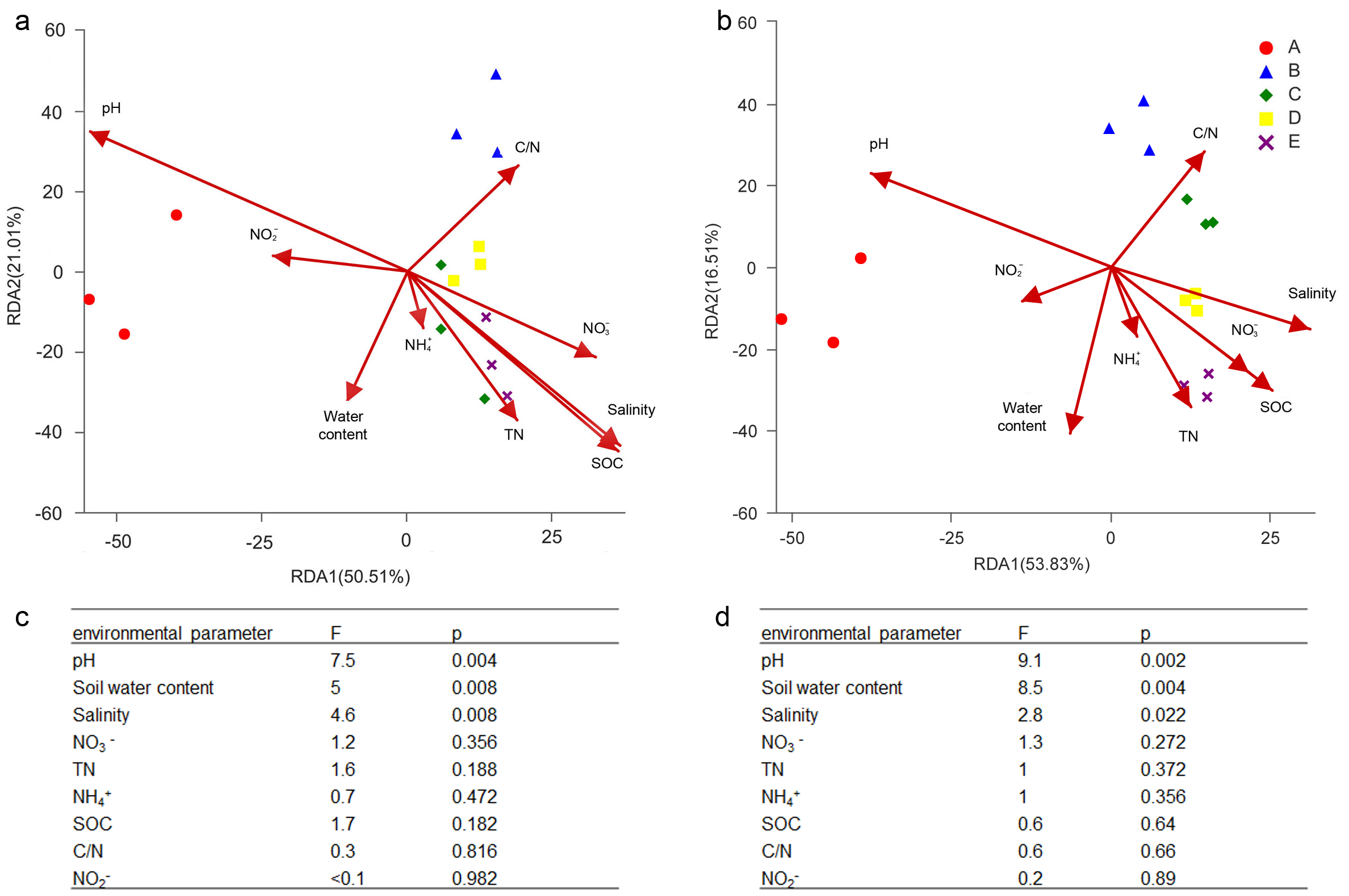

Supplement: FIG S3 [file mSystems.00412-20-sf003.tif]

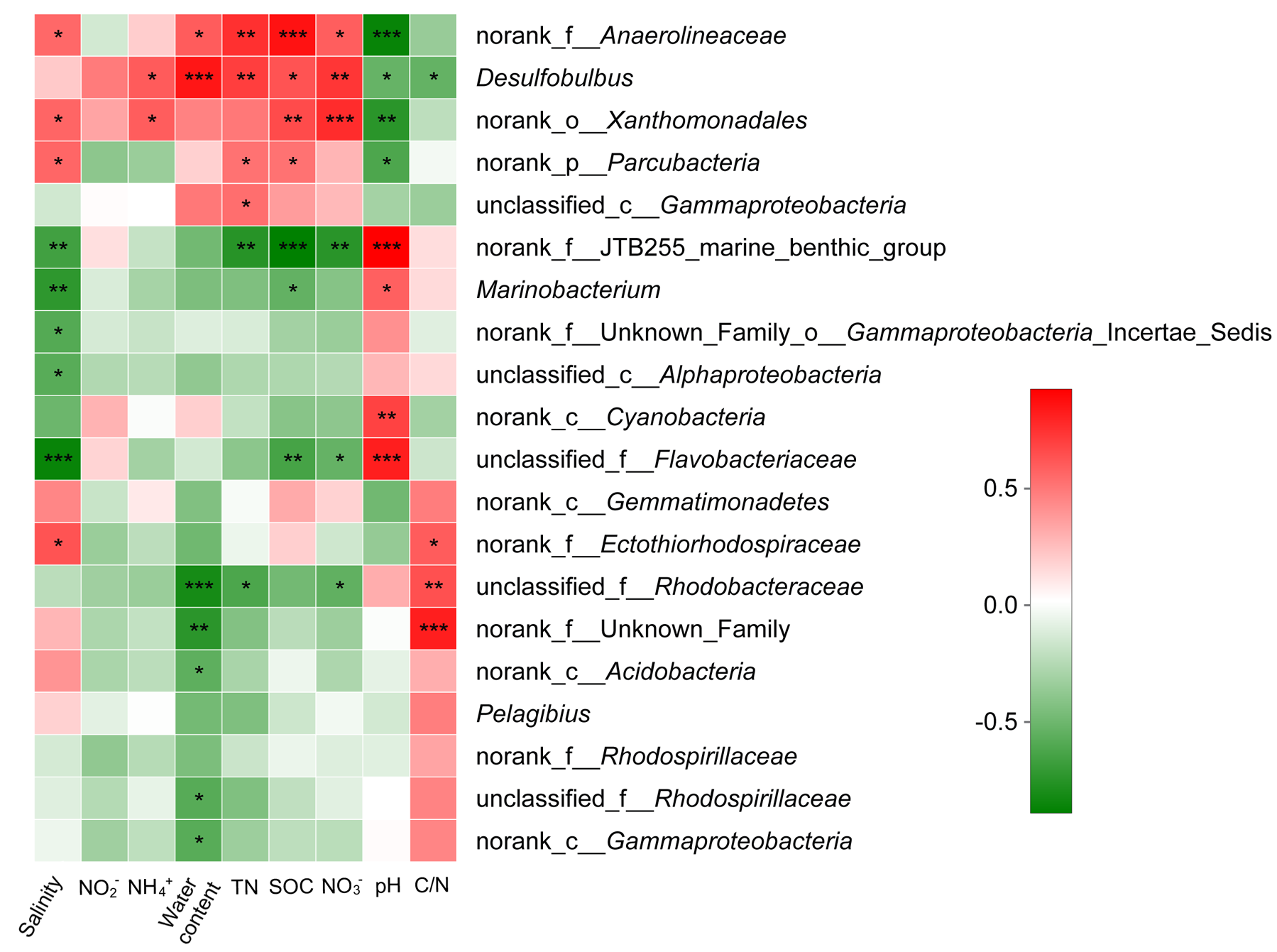

Supplement: FIG S4 [file mSystems.00412-20-sf004.tif]
